# Revolutionizing Medical Imaging: The Transformative Role of Artificial Intelligence in Diagnostics and Treatment

**DOI:** 10.3390/diagnostics15121557

**Published:** 2025-06-18

**Authors:** Daniele Giansanti

**Affiliations:** Centre Tisp, Istituto Superiore di Sanità, 00161 Rome, Italy; daniele.giansanti@iss.it; Tel.: +39-06-49902701

The field of medical imaging is undergoing a significant transformation, driven by the rapid integration of artificial intelligence (AI) into clinical workflows and research methodologies. AI technologies, particularly those based on machine learning and deep learning, have demonstrated immense potential to enhance the precision, efficiency, and personalization of diagnostic and therapeutic processes [1]. These advancements are contributing to a paradigm shift from the traditional, often manual interpretation of medical images to data-driven, automated, and assistive systems that can support clinical decision-making at unprecedented levels [2].

The spectrum of AI applications in medical imaging is growing. Among the most impactful applications are deep learning algorithms designed for image classification and analysis, techniques for robust and accurate image segmentation, and feature extraction methods that can reveal subtle patterns often invisible to the human eye [3]; these innovations have significantly improved the performance of computer-aided diagnosis systems, enabling the earlier and more reliable detection of a wide range of pathologies.

Moreover, AI plays a central role in the prediction of disease progression [4] using longitudinal imaging data, the registration and fusion of multimodal images for more comprehensive assessments, and the development of image-based tools for treatment planning and response evaluation; these tools are increasingly integrated into clinical environments, supporting radiologists, oncologists, and other specialists in tailoring patient-specific interventions [2,3,4].

Alongside these technical advancements, the integration of AI into medical imaging raises important questions about data governance, including issues of data privacy, security, and the ethical use of patient information [5]. Standardizing imaging protocols with AI is also crucial to ensure reproducibility and interoperability across institutions and platforms [6]. Furthermore, the social and ethical implications of adopting AI in healthcare settings requires careful consideration [7], especially in terms of transparency, accountability, and its potential impact on clinical roles and patient trust [8].

Overall, AI is reshaping the landscape of medical imaging, offering powerful tools to support clinicians and improve patient outcomes. At the same time, it calls for a thoughtful and collaborative approach to ensure that technological progress aligns with the fundamental values of medical care.

With the aim of exploring the evolving landscape of research in this field, and following the success of the first edition of this Special Issue (available online at https://www.mdpi.com/journal/diagnostics/special_issues/3FXN9682V0, accessed on 17 April 2025).

We), we decided to launch a second Special Issue intended to serve as a scientific forum for international scholars to meet and exchange ideas (available online at https://www.mdpi.com/journal/diagnostics/special_issues/KN912L0L8Y, accessed on 17 April 2025).

The Special Issue, in addition to this summarizing editorial, includes 20 contributions (contributions 1–20), 15 research articles (contributions 1–15), four reviews (contributions 16–19), and a systematic review (contribution 20).

*Outcomes of the scientific articles*

The analyses conducted in the scientific articles reveal how the integration of artificial intelligence (AI) in medical imaging and diagnostics is revolutionizing healthcare, with a wide array of applications across different specialties. These studies collectively underscore the significant advancements in AI-driven methods for disease detection, prediction, and treatment planning, showcasing how AI is reshaping clinical workflows and enhancing diagnostic accuracy.

In the field of oncology, AI models are improving diagnostic precision. For example, Brunese et al. (contribution 1) presented a U-Net-based deep learning model for liver segmentation in CT and MRI scans, focusing on its explainability and robustness in clinical settings, particularly for hepatocellular carcinoma cases. This method allows for the clearer delineation of liver structures, which is crucial for treatment planning and monitoring. Similarly, Inmutto et al. (contribution 2) explored AI’s ability to differentiate between hepatocellular carcinoma and intrahepatic cholangiocarcinoma using multiphase CT scans, highlighting the model’s performance and interobserver agreement among expert radiologists. The enhanced diagnostic capabilities provided by these AI tools have the potential to significantly improve patient outcomes in liver cancer treatment.

In dermatology, AI applications are making early skin cancer detection more accurate. Gül et al. (contribution 3) introduced a hybrid model combining YOLOv8 and SAM which automates skin lesion detection and segmentation. This approach significantly improves the speed and accuracy of identifying skin cancers such as melanoma, facilitating early intervention and better prognosis for patients.

Regarding prostate cancer diagnostics, Faiella et al. (contribution 4) demonstrated the use of a Random Forest model for predicting lymph node involvement in prostate cancer using mp-MRI data and radiomic features. This method aids in preoperative planning, helping clinicians make informed decisions about treatment strategies. Similarly, Urso et al. (contribution 14) utilized machine learning (ML) models to predict biochemical recurrence in prostate cancer patients following metastasis-directed therapy, based on radiomic features from 18F-choline PET/CT scans. This predictive capability is valuable in tailoring personalized treatment plans and improving patient outcomes.

AI is also transforming the field of cardiology, where it is being applied to enhance diagnostic efficiency. Liu et al. (contribution 6) presented a deep learning model for detecting aortic dissections in CTA scans, specifically Stanford type A and B dissections, a critical condition requiring rapid diagnosis. The model improves diagnostic speed and accuracy, reducing the risk of delayed treatment. In the context of echocardiography, Wu et al. (contribution 7) proposed a UNeXt-based segmentation algorithm for automatic delineation of the left ventricle in transesophageal echocardiography (TEE) images. This tool enhances the precision of cardiac assessments, contributing to improving the diagnosis and management of cardiovascular conditions.

Contributing significantly to cardiac and pulmonary research, Hadhoud et al. (contribution 8) developed a powerful hybrid CNN–ViT framework for the multi-class classification of chest diseases, including tuberculosis and pneumonia, demonstrating that combining convolutional and transformer-based architectures significantly boosts diagnostic accuracy in thoracic imaging.

Dental radiology is another area where AI is having a significant impact. Peker et al. (contribution 5) introduced a YOLOv10-based system for automatic tooth detection and numbering in pediatric panoramic X-rays, offering an efficient solution for pediatric dental care. This AI-driven approach supports better patient management by reducing human error in dental imaging.

Furthermore, AI’s potential in neurological diagnostics is becoming evident. Ianculescu et al. (contribution 9) explored image processing techniques for early Parkinson’s disease detection through hand-drawn-spiral analysis, integrating AI to identify subtle changes that may otherwise go unnoticed. This early-detection capability is crucial for improving long-term patient outcomes by enabling earlier intervention and treatment.

AI’s role extends beyond disease detection into broader clinical applications. For instance, Pellegrino et al. (contribution 10) examined the interaction between ChatGPT-4 and human experts for evaluating Boston Bowel Preparation (BP) Scale scores during colonoscopy, highlighting AI’s growing ability to assist in complex diagnostic assessments. Moreover, Gil-Rios et al. (contribution 11) developed a hybrid metaheuristic algorithm for improving the classification of coronary stenosis from radiological images, further demonstrating AI’s capability in optimizing diagnostic workflows and aiding in precise decision-making.

Additionally, Tian et al. (contribution 12) presented U-Net variants for radial artery tracking in ultrasound images, a tool aimed at supporting catheterization procedures by enhancing the accuracy of artery visualization. This kind of AI application directly contributes to improving procedural accuracy in interventions.

AI’s utility in radiotherapy is also notable. Marquez et al. (contribution 13) analyzed the correlation between dose and geometric metrics in head and neck auto-contouring, underscoring how AI can aid in creating more reliable and effective radiotherapy treatment plans. This work further illustrates AI’s role in optimizing treatment outcomes in oncology.

Lastly, AI is enabling opportunistic screening in other areas, such as vertebral fracture detection. Kim et al. (contribution 15) proposed a deep learning model for screening acute vertebral fractures in routine abdominal and chest CT scans, allowing for opportunistic diagnosis during standard imaging procedures, which could lead to the earlier identification of fractures that might otherwise be missed.

Overall, these studies exemplify how AI is enhancing diagnostic accuracy, accelerating clinical workflows, and providing invaluable support in decision-making across diverse medical specialties. As AI technologies continue to evolve, they will likely become an integral part of clinical practice, improving patient outcomes and transforming healthcare delivery on a global scale.

Table 1 provides a concise overview of the research articles featured in this Special Issue.

*Outcomes of the reviews*

The review studies highlight that he integration of artificial intelligence (AI) with clinical and medical imaging is proving transformative, especially in areas such as interventional radiology, musculoskeletal imaging, cancer diagnostics, and radiotherapy.

As demonstrated by Lastrucci et al. (contribution 16) AI’s application in interventional radiology is rapidly evolving, exhibiting immense potential to revolutionize healthcare by enhancing clinical outcomes and reducing procedural risks. Their review discusses key themes, opportunities, challenges, and future directions, positioning AI as a significant driver of innovation in this field.

Similarly, Mercurio et al. (contribution 17) highlight the promising role of AI in detecting anterior cruciate ligament (ACL) injuries by applying deep learning models applied MRI scans. Their comprehensive review emphasizes the potential of these models to improve diagnostic accuracy and efficiency, highlighting their potential as a new dimension in musculoskeletal imaging.

In the field of oncology, particularly in liver cancer diagnostics, Romeo et al. (contribution 18) explore how AI is shaping the future management of hepatocellular carcinoma (HCC). Their work underscores AI’s growing role in refining diagnostic and predictive models, supporting earlier intervention and more tailored treatment strategies, which are essential given the increasing incidence of HCC.

Lastrucci et al. (contribution 19) further investigate the integration of deep learning in radiotherapy, exploring how this technology is addressing challenges in treatment planning and delivery. Their review emphasizes the acceleration in progress driven by the COVID-19 pandemic, offering valuable insights into the ongoing development and potential of deep learning to optimize radiotherapy procedures.

Lastly, Ramli et al. (contribution 20), in their systematic review, examine AI’s role in lung cancer screening, focusing on its application in detecting lung nodules in chest X-rays. They illustrate AI’s significant contribution to enhancing early lung cancer detection, improving patient prognosis through quicker and more accurate diagnoses.

Overall, these articles underscore AI’s vast potential to improve diagnostic and treatment processes across various medical fields. From interventional radiology to oncology, musculoskeletal imaging, and radiotherapy, AI is reshaping clinical practices, offering new opportunities for more efficient, accurate, and personalized care.

Table 2 provides a concise overview of the review articles featured in this Special Issue.

*Conclusions and future directions*

The integration of artificial intelligence (AI) in medical imaging and diagnostics, as evidenced by the studies (contribution 1–15) and review articles (contribution 16–20) featured in this Special Issue, has been shown to have transformative potential across multiple medical disciplines. These studies collectively underscore AI’s capacity to enhance diagnostic accuracy, streamline clinical workflows, and facilitate personalized treatment strategies, highlighting its role as a driving force in the evolution of healthcare. The findings from the presented research underline the capacity of AI to significantly improve clinical decision-making. For instance, AI-driven models are showing promise in areas like early disease detection, as seen in the diagnostic tools for liver cancer, skin cancer, and prostate cancer. These advancements not only enable more accurate diagnoses but also facilitate faster intervention, which is key to improving patient outcomes. Moreover, AI’s role in enhancing procedural accuracy—whether in radiotherapy or cardiology—highlights its potential to optimize treatment planning and delivery, ultimately contributing to better patient care.

In the future, several key directions will shape AI’s continued integration into healthcare. One important focus will be the refinement of AI algorithms to improve their accuracy, reliability, and explainability. This will be essential to foster trust among clinicians and patients alike, ensuring that AI is seen as a supportive tool rather than a replacement for human expertise. Enhancing AI’s transparency in decision-making processes will be critical for ensuring clinical acceptance and integration. Furthermore, the future of AI in healthcare will depend on the expansion of its capabilities in predictive analytics. Leveraging AI for the early detection and prediction of disease progression can lead to more proactive interventions, potentially reducing the burden of chronic conditions and improving long-term outcomes for patients. As AI models become more sophisticated, they will increasingly support clinicians in making more informed, data-driven decisions that are tailored to individual patient needs. Another important avenue for future exploration will be the continued integration of AI across multi-disciplinary fields. As AI tools become more advanced, their ability to combine data from various imaging modalities and clinical sources will create a more comprehensive picture of patient health. This multi-modal approach can improve diagnostic accuracy, support complex decision-making, and enhance patient outcomes. AI is rapidly becoming an indispensable tool in modern healthcare, offering remarkable potential to improve diagnostic precision, accelerate clinical workflows, and facilitate personalized care. As research continues to advance, AI’s role in healthcare will grow, shaping the future of medical practice and patient care in profound and lasting ways.

## Figures and Tables

**Table 1:** Overview of scientific articles featured in this Special Issue.

Contribution.	Authors	Clinical Area	AI Application
1	Brunese et al.	Liver (Oncology)	U-Net for liver segmentation in CT/MRI; explainability for HCC diagnosis.
2	Inmutto et al.	Liver (Oncology)	Differentiation between HCC and ICC using multiphase CT with AI model.
3	Gül et al.	Skin (Dermatology)	YOLOv8 + SAM hybrid model for skin lesion detection and segmentation.
4	Faiella et al.	Prostate (Oncology)	Random Forest on mp-MRI and radiomics to predict lymph node involvement.
5	Peker et al.	Dental	YOLOv10 system for automatic tooth detection in pediatric panoramic X-rays.
6	Liu et al.	Heart (Cardiology)	Deep learning to detect Stanford A/B aortic dissections in CTA.
7	Wu et al.	Heart (Cardiology)	UNeXt segmentation of left ventricle in TEE images.
8	Hadhoud et al.	Thorax (Heart and Lungs)	CNN–ViT for classification of chest diseases (e.g., TB and pneumonia).
9	Ianculescu et al.	Neurology	AI for early Parkinson’s disease detection from hand-drawn-spiral analysis.
10	Pellegrino et al.	Gastroenterology	Interaction of ChatGPT-4 and experts on Boston Bowel Preparation Scale scoring.
11	Gil-Rios et al.	Cardiology (Coronary)	Metaheuristic hybrid model for classifying coronary stenosis in radiology.
12	Tian et al.	Vascular Intervention	U-Net variants for radial artery tracking in ultrasound.
13	Marquez et al.	Radiotherapy (Oncology)	Correlation of dose/geometric metrics for auto-contouring in head and neck cancer.
14	Urso et al.	Prostate (Oncology)	ML model predicting recurrence in prostate cancer post-therapy using PET/CT radiomics.
15	Kim et al.	Bone/Spine	Deep learning for opportunistic screening of vertebral fractures in CT scans.

**Table 2:** Overview of the review studies featured in this Special Issue.

Contribution.	Authors	Clinical Area	AI Application
16	Lastrucci et al.	Interventional Radiology	Overview of AI integration with interventional radiology, identifying opportunities and challenges.
17	Mercurio et al.	Musculoskeletal (Orthopedics)	Deep learning models for detecting anterior cruciate ligament injuries on MRI.
18	Romeo et al.	Oncology (Hepatology)	AI for managing hepatocellular carcinoma (HCC) through updated diagnostic and predictive models.
19	Lastrucci et al.	Radiotherapy (Oncology)	Deep learning in radiotherapy, exploring its impact on treatment planning and delivery.
20	Ramli et al.	Pulmonology (Lung Cancer)	AI for detecting lung nodules in chest X-rays for early lung cancer screening.
